# Epithelial Heat Shock Proteins Mediate the Protective Effects of *Limosilactobacillus reuteri* in Dextran Sulfate Sodium-Induced Colitis

**DOI:** 10.3389/fimmu.2022.865982

**Published:** 2022-03-07

**Authors:** Hao-Yu Liu, Fang Gu, Cuipeng Zhu, Long Yuan, Chuyang Zhu, Miaonan Zhu, Jiacheng Yao, Ping Hu, Yunzeng Zhang, Johan Dicksved, Wenbin Bao, Demin Cai

**Affiliations:** ^1^College of Animal Science and Technology, Yangzhou University, Yangzhou, China; ^2^Jiangsu Co-Innovation Center for Prevention and Control of Important Animal Infectious Diseases and Zoonoses, Yangzhou University, Yangzhou, China; ^3^Department of Animal Nutrition and Management, Swedish University of Agricultural Sciences, Uppsala, Sweden

**Keywords:** inflammatory bowel disease, barrier function, heat shock proteins, probiotics, gut microbiota

## Abstract

Defects in gut barrier function are implicated in gastrointestinal (GI) disorders like inflammatory bowel disease (IBD), as well as in systemic inflammation. With the increasing incidence of IBD worldwide, more attention should be paid to dietary interventions and therapeutics with the potential to boost the natural defense mechanisms of gut epithelial cells. The current study aimed to investigate the protective effects of *Limosilactobacillus reuteri* ATCC PTA 4659 in a colitis mouse model and delineate the mechanisms behind it. Wild-type mice were allocated to the control group; or given 3% dextran sulfate sodium (DSS) in drinking water for 7 days to induce colitis; or administered *L. reuteri* for 7 days as pretreatment; or for 14 days starting 7 days before subjecting to the DSS. Peroral treatment with *L. reuteri* improved colitis severity clinically and morphologically and reduced the colonic levels of Tumor necrosis factor-α (TNF-α) (*Tnf*), Interleukin 1-β (*Il1β*), and nterferon-γ (*Ifng*), the crucial pro-inflammatory cytokines in colitis onset. It also prevented the CD11b^+^Ly6G^+^ neutrophil recruitment and the skewed immune responses in mesenteric lymph nodes (MLNs) of CD11b^+^CD11c^+^ dendritic cell (DC) expansion and Foxp3^+^CD4^+^ T-cell reduction. Using 16S rRNA gene amplicon sequencing and RT-qPCR, we demonstrated a colitis-driven bacterial translocation to MLNs and gut microbiota dysbiosis that were in part counterbalanced by *L. reuteri* treatment. Moreover, the expression of barrier-preserving tight junction (TJ) proteins and cytoprotective heat shock protein (HSP) 70 and HSP25 was reduced by colitis but boosted by *L. reuteri* treatment. A shift in expression pattern was also observed with HSP70 in response to the pretreatment and with HSP25 in response to *L. reuteri*-DSS. In addition, the changes of HSPs were found to be correlated to bacterial load and epithelial cell proliferation. In conclusion, our results demonstrate that the human-derived *L. reuteri* strain 4659 confers protection in experimental colitis in young mice, while intestinal HSPs may mediate the probiotic effects by providing a supportive protein–protein network for the epithelium in health and colitis.

## Introduction

Complex interactions between the gut microbiota, the epithelial barrier, and the host immune system determine intestinal homeostasis. A breakdown of regulatory pathways in any of these components may lead to chronic inflammatory conditions and inflammatory bowel disease (IBD) (1, 2). Manifestating as Crohn’s disease and ulcerative colitis, IBD affects millions of people worldwide, and the incidence is increasing, especially in the newly industrialized areas, such as Asia (1). Unfortunately, the precise etiology of IBD remains unclear (2). Despite multiple contributors to disease pathogenesis, one common feature of IBD is identified, i.e., the disruption of the intestinal barrier or the so-called leaky gut (3). Animal models (4–6) and clinical studies (2, 7, 8) have revealed that impaired gut barrier function may be an initial event of IBD, allowing bacteria to approach and leak through the mucosa. Consequently, it leads to defective microbial clearance and aberrant immunoregulation and provokes an uncontrollable inflammatory signal cascade in the gastrointestinal (GI) tract and the whole body.

The single layer of intestinal epithelial cells (IECs) represents the largest surface area of human body that is in contact with the external environment. Aside from physically excluding the vast number of luminal bacteria and other noxious agents, IECs also secrete antimicrobial peptides, sense and sample intestinal antigens, and induce immune responses (9). To fulfill such diverse functions, the epithelium forms a selective barrier through complex protein–protein networks (10). Consisting of the tight junction [zonula occludens (ZO)], adherens junction (zonula adherens), and desmosome from an apical to basal direction, it mechanically links adjacent cells, affects the cytoskeleton structure, and seals the intercellular space of IECs (11, 12). Recent studies have demonstrated that the tight junction (TJ) protein expression is altered in IBD patients, while its dysregulation further promotes disease relapse (13). Interestingly, TJ proteins including ZO-1 and occludin show altered expression upon reaction with pathogens (10), as well as with probiotics (4), suggesting bacterial regulatory functions of the intestinal barrier, or vice versa.

The TJ proteins also interact with the actin cytoskeleton and a variety of regulatory elements (14). One such important modulator is the activation of inducible heat shock proteins (HSPs), which protect the gut epithelium against oxidative stress and inflammation (15). As highly conserved molecular chaperones, HSPs carry out fundamental gatekeeping functions including import/translocation of proteins into appropriate cellular compartments, folding and refolding of misfolded proteins, degradation of unstable proteins, and control of regulatory proteins for maintenance of cell homeostasis (15, 16). More importantly, heat responses to cell injury and allelic polymorphisms in genes encoding HSP70 have been reported in IBD patients (17). While in aging people with compromised immunity, HSP70 is downregulated, concomitant with increased levels of pro-inflammatory cytokines in circulation (18). Furthermore, we and others have revealed an axial gradient of HSP25/27 and HSP70 expression in the mucosa, aligned with the microbiota density and diversity in the mammalian GI tract (15, 19). In contrast, antibiotic-treated mice exhibit a significant reduction of HSP25 and HSP70 expression in the colon (20), suggesting bacterial regulatory functions of epithelial HSPs, or vice versa.

Although no approved therapeutics that target the epithelial barrier are currently available, approaches specifically regulate the TJ, and unrestricted pathways are in progress, including probiotic treatment (12, 21). Members of the diverse Lactobacillus taxon have been rigorously tested as probiotics (live microorganisms when given in adequate amount confer health benefits on the host) in animal models of GI diseases (22). Oral administration of Lactobacillus rhamnosus GG is shown to reduce colitis and diarrhea (23). Our previous studies demonstrate that peroral treatment with Limosilactobacillus reuteri strengthens the intestinal barrier by upregulating TJ protein expression (4) and improves the gut microbiota–IgA interactions, thereby protecting against colitis in mice (24).

Nevertheless, to embody the scientific observations for clinical use, a deeper understanding of IBD pathogenesis and mechanisms of barrier regulation must be achieved. In the current study, we determined the role of major inducible HSP70 and HSP25 in the pathogenesis and probiotic treatment of IBD using a dextran sulfate sodium (DSS)-induced colitis model in young mice. We found that the expression of cytoprotective HSPs was reduced by colitis that also coincided with changes in TJ protein expression, inflammatory responses, and gut microbiota dysbiosis. This HSP-mediated effect was also regulated by peroral treatment with *L. reuteri* ATCC PTA 4659, a human-derived probiotic strain. We, therefore, propose that epithelial HSPs may make up a potential target for the treatment of IBD.

## Materials and Methods

### Mice and Dextran Sulfate Sodium-Induced Colitis Model

Male C57BL/6J wild-type mice were purchased from the Jiangsu Laboratory Animals Science Center and were housed at 22°C with 50% humidity on a 12/12-h light/dark cycle and allowed free access to water and a standard chow diet. The animals were subjected to experiments at 10 weeks old under guidelines of the Animal Care and Use Committee of the Yangzhou University (YZUDWSY 2017-09-06). Mice were randomly divided into four different groups (n = 6–8): control, pretreatment with *L. reuteri* (*L. reuteri*), DSS-treated (DSS), and the cotreatment with *L. reuteri* and DSS (*L. reuteri*-DSS). To induce colitis, 3% DSS in drinking water was administered to mice for 7 days, where the disease activity of colitis was examined daily and presented as a disease activity index (DAI) with a maximum score of 4 as described previously (4). In brief, DAI was calculated based on the percentage of weight loss from initial body weight, stool consistency (normal, loose, or diarrhea), and rectal bleeding using hemoccult test (25). For probiotic bacteria groups, *L. reuteri* ATCC PTA 4659 was freshly prepared and was given perorally at 10^8^ CFU per mouse per day for 7 consecutive days (*L. reuteri*) or for 14 days starting 7 days prior to the DSS treatment (*L. reuteri*-DSS).

### Collection of Samples

At the end of each experiment, the mice were anesthetized by intraperitoneal injection of 250 μl of 0.9% sodium pentobarbital solution, and blood was collected by cardiac puncture with Ethylenediaminetetraacetic acid (EDTA) collection tubes. The terminally anesthetized mice were then euthanized by cervical dislocation. Macroscopy analysis was performed upon dissection on changes of GI length and spleen and mesenteric lymph node (MLN) sizes. For bacterial analysis, luminal contents from the distal ileum and the distal colon and MLNs were collected, immediately frozen in liquid nitrogen, and stored at −80°C until DNA isolation. Mucosa samples from each location were taken and divided for RNA extraction, histological and immunohistochemical studies, and protein measurements. In addition, fresh MLN samples and the blood were immediately subjected to flow cytometry analysis.

### Measurement of Cytokines and Barrier Proteins

The cytokine expression was measured in the distal colonic tissues including Il10, Il1β, Il6, Ifng, and TNF-α (Tnf). Total RNA was extracted using TRIzol reagent. The cDNA was prepared, amplified, and measured in the presence of SYBR Green (Thermo Fisher Scientific) as previously described (24). Briefly, the fluorescent values were collected, and a melting curve analysis was performed. Glyceraldehyde-3-Phosphate Dehydrogenase (Gapdh) was used as the internal reference for normalization. Relative expression of target genes was determined by 2^−ΔΔCt^ method. The primer sequences are shown in **Supplementary Table S1**. Next, the production of pro-inflammatory cytokine TNF-α, important for IBD pathogenesis (26), was assessed by ELISA according to the manufacturer’s instructions (Mouse TNF-alpha Quantikine ELISA Kit, R&D Systems). Values were normalized to tissue protein contents measured by Bovine Serum Albumin (BCA) methods.

### Flow Cytometry

Single-cell suspensions from MLNs were prepared by mashing organs through 40-µm cell strainers in Phosphate Buffered Saline (PBS) containing 0.05% Fetal Bovine Serum (FBS) and 2 mM EDTA (Fluorescence-activated cell sorting (FACS) buffer). Whole-blood samples were directly processed with a standardized protocol. Samples were incubated in a red blood cell lysis buffer (150 mM NH_4_Cl, 10 mM KHCO_3_, 0.1 mM EDTA) for 10 min at room temperature. The reaction was then stopped with an equal amount of PBS. After centrifugation, cells were resuspended in FACs buffer. Thereafter, single-cell suspensions were incubated with Fc blocker (2.4G2) from BD Biosciences. The cell-surface staining was then performed using the following antibodies: anti-CD11b (M1/70), Ly6G (1A8), CD3 (17A2), and CD4 (GK1.5) antibodies from BioLegend and anti-CD11c antibody (N418) from eBioscience. Dead cells were excluded from analysis using the Live/Dead Aqua Viability Kit (Thermo Fisher Scientific). For intracellular staining, cells were fixed and permeabilized with FoxP3 Staining Buffer Kit according to manufacturer’s instructions and then stained with anti-Foxp3 (FJK-16s) antibody from eBioscience. Data were acquired on an LSR Fortessa SORP flow cytometer (BD Biosciences) and analyzed with FlowJo version 10.0.8 (Tree Star, Inc.).

### Histological Analysis and Immunohistochemistry

Tissues obtained from the ileum and distal colon were routinely fixed with 4% paraformaldehyde overnight, dehydrated in 70% ethanol, embedded in paraffin, sectioned (5 μm in thickness), and stained with H&E for histological analysis. Subsequently, the ileal villus height and crypt depth were assessed, where the ratio of the two parameters (V:C) was calculated. In the distal colon, the total mucosal thickness was measured (n = 5 per group, two slides per mouse) and imaged with a light microscope (Leica DFC 420C).

For immunohistochemistry, the intestinal tissues were placed in Tissue-Tek OCT and snap-frozen in liquid nitrogen. Thereafter, the samples were cryosectioned (15 µm) and stained for target proteins. Antibodies raised against the following mouse antigens were used: ZO-1 (ABIN602576, antibodies-online), occludin (ABIN1108503, antibodies-online), HSP70 (ADI-SPA-812, Enzo), HSP25 (ADI-SPA-801, Enzo), Ki67 (ab15580, Abcam), and Phalloidin-Alexa Fluor 555 (A34055, Thermo Fisher Scientific). Accordingly, secondary antibodies were used to amplify signals with Alexa Fluor 488 anti-rat, Alexa Fluor 647 anti-guinea pig, and Alexa Fluor 488 anti-rabbit IgG. Nuclei were stained with Hoechst 33342 (Thermo Fisher Scientific). Two slides of each mouse (n = 6 per group) were imaged using a Zeiss confocal Laser Scanning Microscope 780. For HSP70 and HSP25, mean fluorescence intensity (MFI) was quantified in the distal colon as upper half of mucosal surface and lower half of crypt, respectively. And the number of Ki67^+^ cells per villi-crypt unit in the ileum and colon was counted using ImageJ software (Rasband, W.S., ImageJ, U.S. National Institutes of Health, Bethesda, MD, USA, https://imagej.nih.gov/ij/, 1997-2018).

### Western Blotting

Western blot analysis of the distal colonic mucosal samples was performed by Sodium dodecyl-sulfate polyacrylamide gel electrophoresis (SDS-PAGE) and transferred to membrane. For the detection, primary antibodies against HSP70 (ADI-SPA-812), HSP25 (ADI-SPA-801), and HSC70 (ADI-SPA-815, Enzo) and the corresponding secondary antibodies (goat anti-rabbit IgG HRP, sc-2004; or goat anti-rat IgG HRP, dv-2006, Santa Cruz Biotechnology) were used. Proteins were visualized using chemiluminescent detection reagent (Thermo Fisher Scientific, MA, USA) and analyzed using the FluorChem FC3 Chemiluminescent system (ProteinSimple Ltd., USA).

### Extraction of DNA and Analysis of Gut Microbiota

DNA was extracted from the ileal and colonic luminal contents (180–220 mg) using QIAmp DNA Stool Mini kit and from MLNs using DNA Mini kit (Qiagen) according to the manufacturer’s instructions. The bacterial load was quantified by RT-qPCR with primers encoding the bacterial V3-V4 16S rRNA gene (**Supplementary Table S1**) and normalized with sample weight.

Thereafter, gut microbiota composition was characterized by 16S rRNA gene sequencing as described previously (24). Briefly, the bacterial V3-V4 16S rRNA gene regions were PCR amplified from each sample using a composite forward primer and a reverse primer containing a unique 8-base index primer, designed to tag PCR product from respective samples (**Supplementary Table S1**). PCR reactions consisted of a master mix [0.5 µl of each primer, 4 µl of Q5 reaction buffer, 0.2 µl (0.02 U/µl) Q5 HF DNA polymerase (New England Biolabs), 2 µl of dNTPs, 11.8 µl of Milli-Q water] and 1 µl of DNA template. Reaction conditions were 1 min at 98°C, followed by 20 cycles of 10 s at 98°C, 30 s at 58°C, 30 s at 72°C, and a final extension step at 72°C for 2 min on a Bio-Rad thermocycler. Duplicates were run in 20-μl reactions for each sample, combined, and purified with Agencourt Ampure magnetic purification beads (Beckman Coulter). Then, a second PCR was conducted using 1:10 dilutions of the first PCR product for attaching standard Illumina handles and index primers. The steps were the same as mentioned above with modifications (18 thermal cycles and the annealing temperature at 63°C). PCR products were quantified with Picogreen dsDNA assay according to the manufacturer’s instruction (Thermo Fisher Scientific) and sequenced on an Illumina HiSeq 2500 sequencer. The Illumina sequencing data output was processed according to the cutoffs and pipeline described previously (27). Sequences were assigned to operational taxonomic units (OTUs) by using a closed reference-based OTU picking method in QIIME v1.8. For every OTU, the sequence was checked as a query against the SILVAMOD database using the CREST software version 2.0. For comparative measurements of total bacterial load, a primer for total 16S rRNA primers was used. The data were normalized by sample weight.

### Statistical Analysis

Statistical analysis was performed using GraphPad Prism software v.9.0. Two-tailed Student’s t test was used for direct comparison of two groups and ANOVA with Tukey’s *post-hoc* test to compare all groups. Difference of body weight change was compared as area under the curve. Pearson correlations of the bacterial loads, parameters of immune responses, and the expression of HSPs in the colon within animal were conducted, and the Pearson correlation (r) of the means of each measurement was calculated. For microbial community composition analysis, bioinformatics was performed using the cumulative-sum scaling method (28). VEGAN package version 2.0-7 and the R statistical framework version 2.11 were used to perform multivariate analysis of microbiota; α-diversity is represented by Shannon index. Data were presented as mean ± SEM; *p* < 0.05 was considered significant. Statistical details and the exact value of “n” can be found in the Figure legends.

## Results

### Peroral Treatment with *Limosilactobacillus reuteri* Reduces Inflammatory Responses and Confers Protection Against Dextran Sulfate Sodium-Induced Colitis

**Figure 1:**
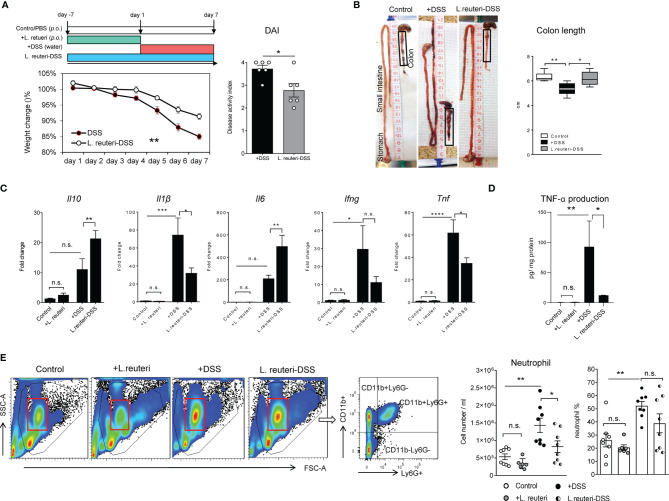
Peroral treatment with *Limosilactobacillus reuteri* reduces inflammatory responses in DSS-induced colitis in young mice. **(A)** Wild-type mice were administered 3% DSS in drinking water for 7 consecutive days (red band); *L. reuteri* ATCC PTA 4659 (10^8^ CFU) was given daily for 7 days (green band) or 14 days starting 7 days prior to DSS treatment (*L. reuteri*-DSS, blue band) as illustrated. Body weight was recorded daily, and disease activity index (DAI) was calculated. **(B)** Representative images show changes of intestinal morphology and colon length (cm). **(C)** q-RT-PCR analysis of gene expression of cytokines (fold change) and **(D)** TNF-α production (pg/mg protein). **(E)** Flow cytometry analysis of neutrophil (live CD11b^+^Ly6G^+^) responses in blood. Data are presented as mean ± SEM (n = 6–8 mice per group). *p < 0.05, **p < 0.01, ***p < 0.001, ****p < 0.0001 using two-tailed Student’s t test and ANOVA with Tukey’s *post-hoc* test. DSS, dextran sulfate sodium. ns, non significant.

Here, we studied the protective effects of *L. reuteri* ATCC PTA 4659 by employing a chemical model of colitis with 3% DSS administration in young mice. When compared to the DSS-only group, reduced weight loss (**Figure 1A**, left panel, *p* < 0.01), DAI (**Figure 1A**, right panel, *p* < 0.05), and colon shortening (**Figure 1B**, *p* < 0.05) were observed in the *L. reuteri*-DSS group. Furthermore, analyses of major cytokines revealed that DSS increased the expression of *Il1β*, *Ifng*, and TNF-α (*Tnf*) without affecting *Il10* or *Il6* levels in the distal colon of mice compared to the control (**Figures 1C, D**; *p* < 0.05). By contrast, the expression of *Il1β*, *Ifng*, and TNF-α (*Tnf*) was significantly decreased by *L. reuteri* treatment during DSS-induced colitis (*p* < 0.05) without affecting *Il6* or *Il10* levels (**Figures 1C, D**; *p* > 0.05). Notably, pretreatment with *L. reuteri* for 7 consecutive days did not induce any inflammatory responses with regard to cytokine expression when compared to the control group (*p* > 0.05). Finally, as the hallmark of acute colitis, the neutrophil recruitment was examined (4). Flow cytometry analysis showed that DSS promoted the expansion of CD11b^+^Ly6G^+^ neutrophils in blood with increased numbers and percentage compared to the control (**Figure 1E**, *p* < 0.01). The cell number increase was diminished by *L. reuteri* treatment during colitis (**Figure 1E**, *p* < 0.05), indicating less neutrophil recruitment to the effector sites. No significant difference was found between the pretreatment group and the control group (**Figure 1E**, *p* > 0.05).

**Figure 2:**
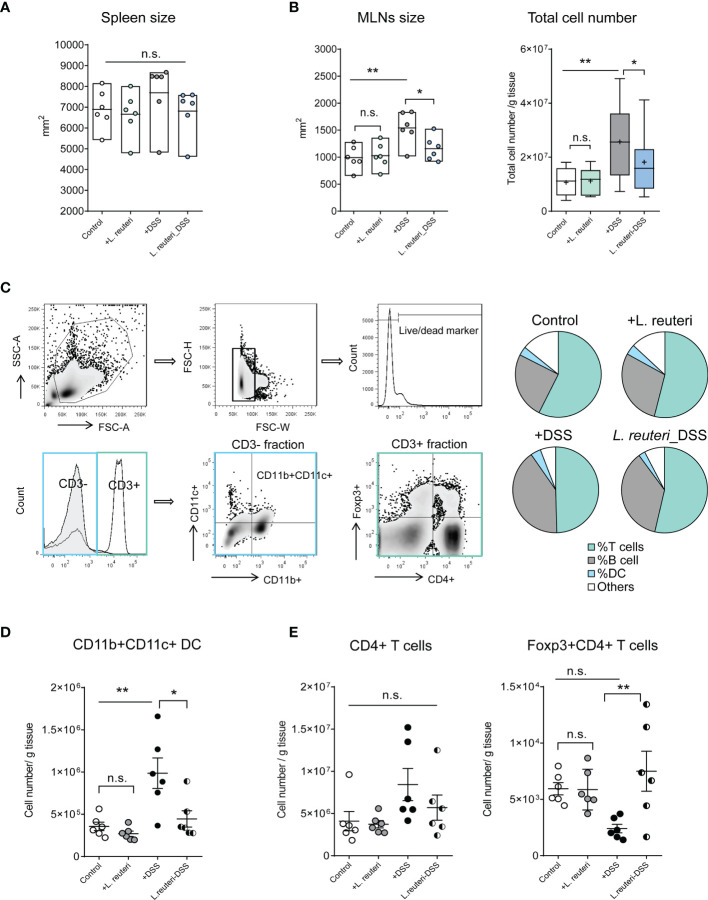
Effects of *L. reuteri* treatment on lymphoid tissues in DSS-induced colitis in young mice. **(A, B)** Macroscopic analysis of spleen and mesenteric lymph nodes (MLNs) measured for size, as well as the total cell number (number/g tissue) in MLNs. **(C–E)** The gating strategy of cell subsets isolated from MLNs in flow cytometry analysis (upper panel) and the relative proportion of immune cells in MLNs (lower panel, **C**). MLN accumulation of live CD3^-^CD11b^+^CD11c^+^ dendritic cells **(D)** and CD4^+^ T cells **(E)**. Data are presented as mean ± SEM, n = 6 mice per group. *p < 0.05, **p < 0.01 using ANOVA with Tukey’s *post-hoc* test. DSS, dextran sulfate sodium. ns, non significant.

These observations prompted us to investigate the effects of probiotic bacteria on secondary lymphoid tissues in mice, especially the MLNs. Peroral administration of *L. reuteri* did not change the size of spleen in health or colitis (**Figure 2A**, *p* > 0.05). However, it preserved the enlargement of MLNs and the cell accumulation in MLNs caused by DSS (**Figure 2B**, *p* < 0.05). Flow cytometry analysis revealed that *L. reuteri* treatment restored the distribution of immune cells in MLNs during colitis into a similar profile as the control group (**Figure 2C**). In particular, *L. reuteri* treatment reduced the number of CD11b^+^CD11c^+^ dendritic cells (DCs) (**Figure 2D**, *p* < 0.05), a major source of pro-inflammatory cytokines during colitis, whereas the number of Foxp3^+^CD4^+^ T cells in MLNs was increased compared to the DSS-only group (**Figure 2E**, *p* < 0.001). Again, pretreatment with *L. reuteri* did not stimulate overt responses in MLNs with regard to immune cell populations when compared to that in the control group (*p* > 0.05). Together, these results demonstrate that *L. reuteri* treatment ameliorates inflammation and the associated immune reactions, conferring protection against DSS-induced colitis in mice.

### *Limosilactobacillus reuteri* Results in Important Changes in Gut Microbial Ecology During Colitis in Mice

**Figure 3:**
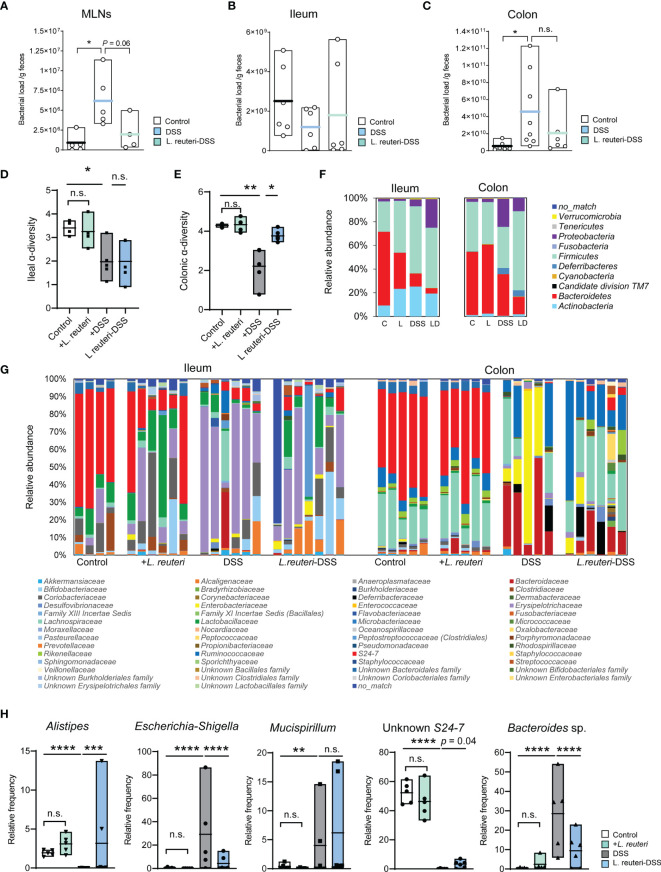
Peroral treatment with *L. reuteri* reduces gut microbiota dysbiosis in DSS-induced colitis in young mice. **(A–C)** Microbial loads in MLNs and ileal and colonic luminal contents were quantified by q-RT-PCR on the 16S rRNA gene (normalized to sample weight). **(D, E)** Ileal and colonic microbiomes were assessed by 16S rRNA gene amplicon sequencing. The α-diversity of microbial community was calculated. **(F, G)** Taxonomic distributions of bacterial composition in the ileum and colon at phylum and family levels. **(H)** Relative abundance of *Alistipes*, *Escherichia-Shigella*, *Mucispirillum*, Unknown *S24-7*, and *Bacteroides* sp. in the colonic microbiota (n = 4–6 mice per group). C, control; L, *L. reuteri*; D, LD, *L. reuteri*-DSS. Data are mean ± SEM. *p < 0.05, **p < 0.01, ***p < 0.001, and ****p < 0.0001 (*p*-values were adjusted with false discovery rate control using the method of Benjamini and Hochberg). DSS, dextran sulfate sodium, MLNs, mesenteric lymph nodes. ns, non significant.

Bacterial antigens are recognized and transported from the intestine by DCs to the MLNs, which may be deployed to exacerbate inflammation in colitis mice (5). Indeed, we found that DSS increased the bacterial translocation in MLNs using RT-qPCR of the 16S rRNA genes (**Figure 1A**, *p* < 0.05). *L. reuteri* treatment minimized the bacterial load in MLNs to a similar level as in the control group (**Figure 3A**, *p* = 0.05). The microbial load was also measured in the ileal and colonic luminal contents in mice. A trend of decrease was observed in the ileum in DSS-associated mice (**Figure 3B**, *p* > 0.05), whereas an opposite and significant response was found in the colon (**Figure 3C**, p < 0.05).

Next, we determined the effect on the gut microbiota by 16S rRNA gene amplicon sequencing. DSS-induced colitis strongly reduced the α-diversity of both ileal (**Figure 3D**, p < 0.05) and colonic bacterial community (**Figure 3E**, p < 0.01) compared to the healthy mice from the control group and the pretreatment with *L. reuteri*. Peroral treatment with *L. reuteri* preserved the microbiota diversity in the colon (p < 0.05) but not in the distal ileum (p > 0.05). These changes may attribute to the relative abundance reduction of the most abundant phylum Bacteroidetes, particularly the relative abundance reduction of *S24-7* at the family level (affiliated with Bacteroidetes) in the ileum in response to DSS and/or *L. reuteri* (**Figures 3F, G**, left panel). In contrast, the relative abundance of the Erysipelotrichaceae (a family affiliated with Firmicutes) in the ileum was increased by the DSS treatment compared with the control mice (p < 0.05), while its relative abundance was not restored to the pre-DSS treatment level by peroral treatment with *L. reuteri* (**Figure 3G**, p > 0.05). In the colon, *L. reuteri* treatment resulted in significant gut microbiota composition alteration against DSS-induced colitis (**Figures 3F, G**, right panel). The relative abundance of the bacterial taxa *Alistipes* (a genus affiliated with *Rikenellaceae*) and an unknown *S24-7* was diminished by DSS treatment (**Figure 3H**, p < 0.0001). By contrast, the relative abundance of *Bacteroides* sp. was significantly increased by DSS compared to that in the control (**Figure 3H**, p < 0.0001). When compared with the DSS-only group, *L. reuteri* treatment altered the abundance of these three bacterial taxa belonging to the Bacteroidetes phylum (**Figure 3H**, p < 0.05). Moreover, Escherichia-Shigella (affiliated with Enterobacteriaceae) and Mucispirillum (affiliated with Deferribacteraceae) were identified as the major pathobionts in the colon, with their relative abundance increased from less than 2% in healthy mice to up to 80% and 15% in DSS-induced colitis, respectively (**Figure 3H**, p < 0.01). *L. reuteri* treatment inhibited the overgrowth of Escherichia-Shigella (**Figure 3H**, p < 0.0001) but not Mucispirillum (p > 0.05). Notably, *L. reuteri* treatment changed the bacterial community during colitis into an alternative state, different from the DSS or the control group (**Figures 3F–H**). Nevertheless, these results suggest that *L. reuteri* treatment improves the DSS-disrupted gut microbial ecology, especially in the colon.

### *Limosilactobacillus reuteri* Induces the Expression of Tight Junction Proteins and Intestinal Heat Shock Proteins

**Figure 4:**
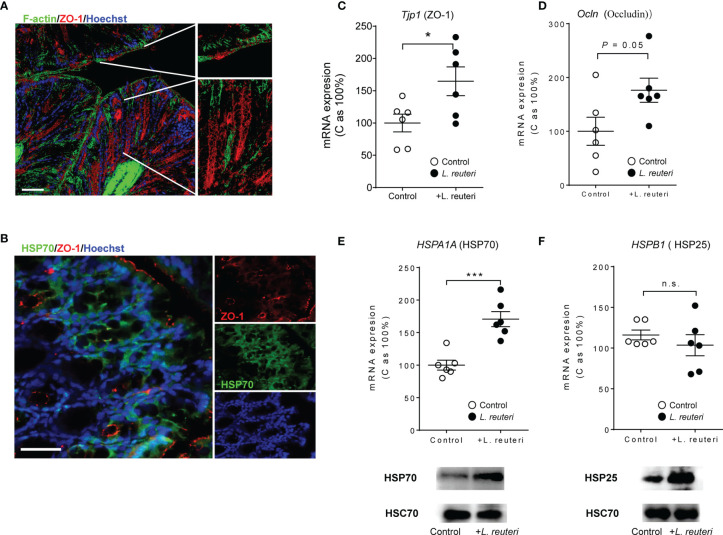
Detection of colonic tight junction proteins and heat shock proteins (HSPs) in response to *Limosilactobacillus reuteri* in healthy young mice. 10 week-old wild-type mice were given *L. reuteri* ATCC PTA 4659 (10^8^ CFU) daily for 7 consecutive days. Confocal images of colonic tissues stained with anti-ZO-1 (red), phalloidin (Green), and Hoechst (blue, **A**) and anti-HSP70 antibodies (Green, **B**). Scale bar = 50 μm. Expressions of tight junction proteins **(C, D)** and HSP70 and HSP25 **(E, F)** were measured by q-RT-PCR and immunoblotting. Data are presented as mean ± SEM, n = 6 mice per group. *p < 0.05, ***p < 0.001 using two-tailed Student’s t test. DSS, dextran sulfate sodium. ns, non significant.

The expression of TJ protein, F-actin, and HSP70 was assessed by immunohistochemistry and confocal microscopy in the distal colon (**Figures 4A, B**). In consistency with our previous findings of probiotic-induced TJ protein expression (4), pretreatment with *L. reuteri* upregulated the expression of Tjp1 (encoding ZO-1, **Figure 4C**, p < 0.05) and Ocln (encoding occludin, **Figure 4D**, p = 0.05) when compared to that of the control group. Similarly, it enhanced the expression of two major inducible HSPs, i.e., HSP70 (HSPA1A, **Figure 4E**, p < 0.001) and HSP25 (HSPB1), in the distal colon of mice at mRNA and/or protein levels when compared to that of the control animals (**Figures 4E, F**).

### *Limosilactobacillus reuteri* Alters Intestinal Heat Shock Protein Expression Pattern and Protects Barrier Function During Colitis

**Figure 5:**
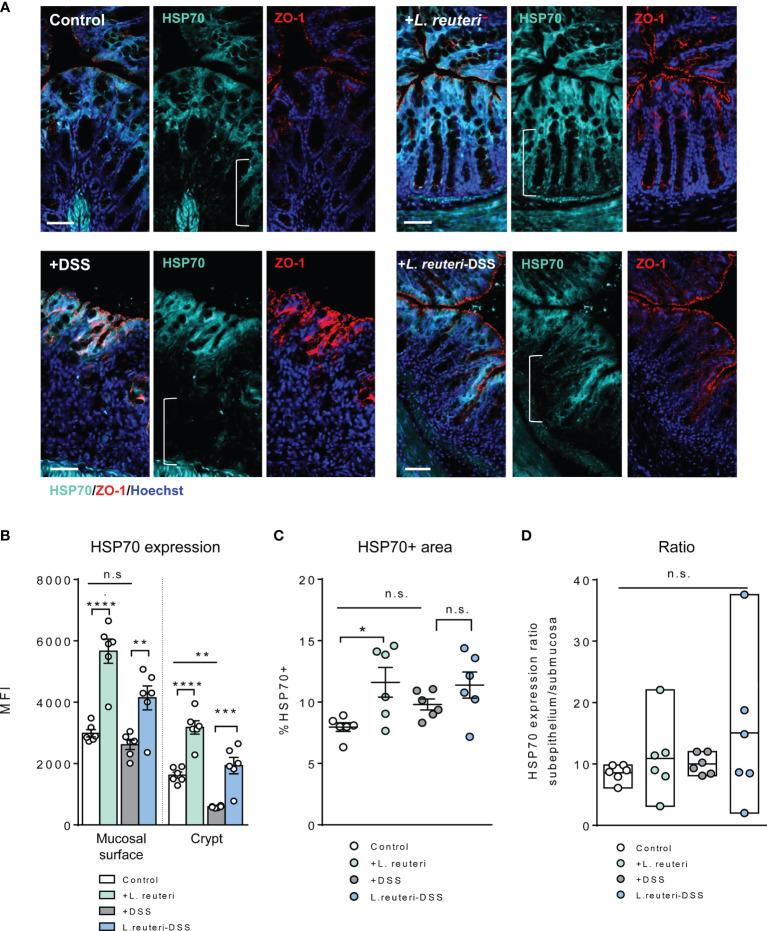
Alterations of HSP70 expression in the colon in response to *L. reuteri* during DSS-induced colitis in young mice. **(A)** Representative images of colonic tissue sections stained with antibodies specific for HSP70 (cyan) and ZO-1 (red). Sections were counterstained with Hoechst (blue). Scale bar = 50 μm. **(B–D)** Analysis and quantification of HSP70 expression, n = 6 mice per group (duplicate slides per mouse). Data are presented as mean ± SEM. *p < 0.05, **p < 0.01, ***p < 0.001, and ****p < 0.0001 using ANOVA with Tukey’s *post-hoc* test. DSS, dextran sulfate sodium. ns, non significant.

**Figure 6:**
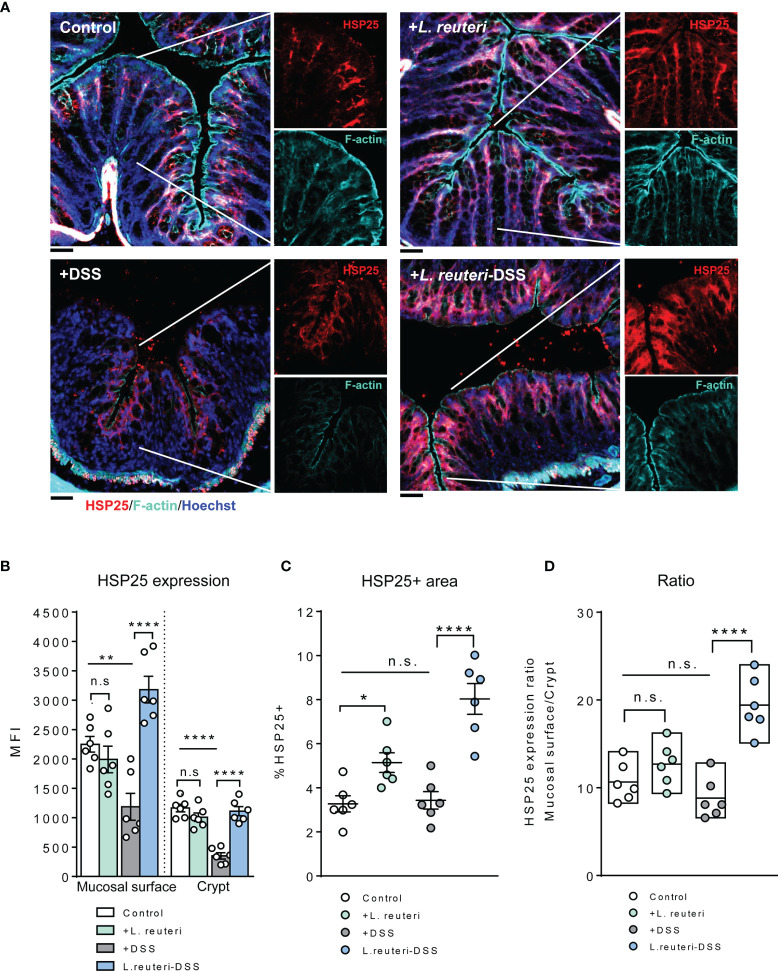
Alterations of HSP25 expression in the colon in response to *L. reuteri* during DSS-induced colitis in young mice. **(A)** Representative images of colonic tissue sections stained with anti-HSP25 (red) and phalloidin (cyan). Sections were counterstained with Hoechst (blue). Scale bar = 50 μm. **(B–D)** Analysis and quantification of HSP25 expression, n = 6 mice per group (duplicate slides per mouse). Data are presented as mean ± SEM. *p < 0.05, **p < 0.01, ****p < 0.0001 using ANOVA with Tukey’s *post-hoc* test. DSS, dextran sulfate sodium. ns, non significant.

We further assessed the HSP expression associated with gut barrier injury during DSS-induced colitis (**Figures 5**, **6**). Firstly, HSP70 was mainly localized to the surface mucosa of the distal colon in healthy control, whereas the lower half of the crypt was almost lacking its expression (**Figures 4B**, **5A**). Furthermore, pretreatment with *L. reuteri* increased the MFI of HSP70 in both surface mucosa and the crypt (**Figures 5A, B**), as well as expanded its distribution, when compared to that of the control mice (**Figure 5C**, p < 0.0001). In contrast, DSS treatment disrupted the colonic barrier integrity and further reduced the crypt HSP70 levels when compared to those of the control (**Figures 5A, B**; p < 0.01). In the *L. reuteri*-DSS group, the pattern of HSP70 expression was completely restored, the same as that observed in healthy control mice, along with the normalized mucosa morphology (**Figures 5A–D**, p < 0.05).

A similar tendency was observed for HSP25 expression. Pretreatment with *L. reuteri* expanded its distribution in the colon, attributed to a significant increase in the lower half of the crypt, compared to that in the control (**Figures 6A–C**, p < 0.05). No significant difference was detected with the MFI of HSP25 (**Figure 6B**, p > 0.05). Interestingly, it seemed that *L. reuteri* treatment had a stronger impact on the HSP25 expression during DSS-induced colitis than that of HSP70, i.e., DSS treatment resulted in significantly lower MFI of HSP25 in both the surface mucosa and the lower half of the crypt in the distal colon. While *L. reuteri* treatment maintained these changes during colitis, it specifically enhanced the HSP25 responses in the surface mucosa (**Figure 6A**), reflected by the increased MFI, the HSP25-positive area, and the significantly increased mucosal surface/crypt expression ratio compared to those of the DSS-only group (**Figures 6A–D**, p < 0.0001).

### *Limosilactobacillus reuteri* Alters the Epithelium Proliferation and the Surrounding Tissues

**Figure 7:**
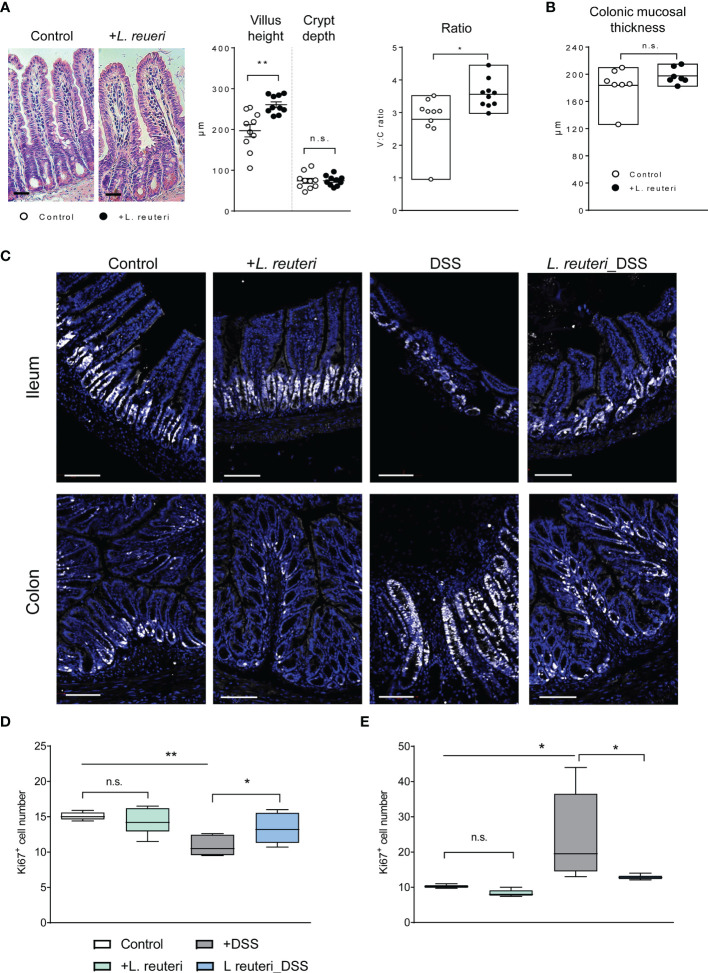
Alterations of intestinal histological parameters and proliferation in response to *L. reuteri* during DSS-induced colitis in young mice. **(A)** Representative images of ileum of mice. Villus height and crypt depth were measured, and the ratio V:C was calculated, as well as the mucosal thickness of the colon **(B)**. **(C–E)** Analysis and quantification of proliferating cells. **(A)** Representative staining of ileum (upper panels) and colon (lower panels) with anti-Ki67 (white) and Hoechst (blue). The number of Ki67^+^ cells per villi-crypt unit was measured (n = 6 mice per group, two slides per mouse). Scale bar = 50 µm. Data are presented as mean ± SEM. *p < 0.05, **p < 0.01 using ANOVA with Tukey’s *post-hoc* test. DSS, dextran sulfate sodium. ns, non significant.

Finally, we studied epithelial cell proliferation and their spatial organization along the crypt axis in health and colitis (**Figure 7**). Histological analysis with H&E staining showed that pretreatment with *L. reuteri* increased the ileal villus height (**Figure 7A**, p < 0.01) and the V:C ratio (p < 0.05), but not the crypt depth or the colonic mucosal thickness (p > 0.05), indicating an increased absorption area in the intestine of these mice. Epithelial cells along the crypt axis in healthy mice are renewed every 3–5 days through cell proliferation, we therefore stained the intestinal tissues with the proliferative marker Ki67. DSS treatment reduced the number of Ki67-positive cells in the ileum (**Figures 7C–E**, p < 0. 01) but increased it in the colon significantly compared to the control group (p < 0.05). At each site, *L. reuteri* treatment preserved the changes against colitis to that of the control levels (**Figures 7C–E**, p < 0.05). It is worth mentioning that villi blunting and ileal atrophy were observed in the DSS-only group (**Figure 7C**), as well as demonstrated in our previous studies (24). The difference of DSS-induced cell proliferation in the ileum and colon may also be linked with the divergent effects of DSS on microbiota.

**Figure 8:**
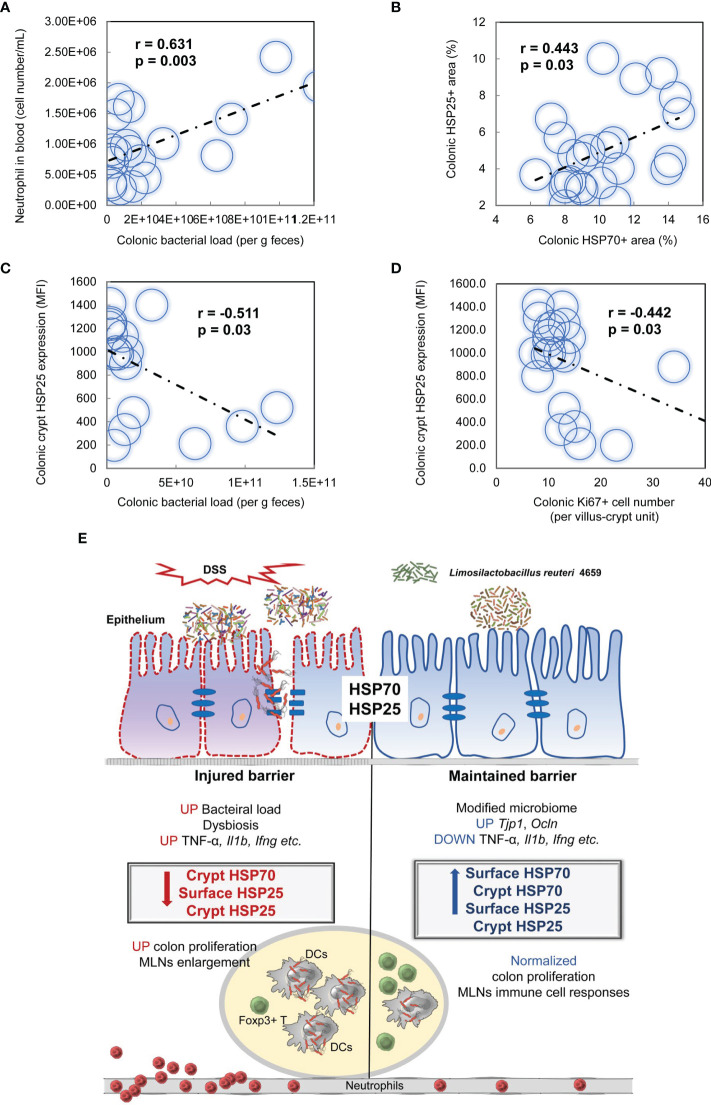
Pearson correlations of the bacterial loads, parameters of immune responses, and the expression of heat shock proteins (HSPs) in the colon within animals were conducted in response to *L. reuteri* during DSS-induced colitis in young mice (**A–D**, n = 24). **(E)** Schematic illustration of epithelial HSP-mediated protective effects of *L. reuteri* ATCC PTA 4659 during established colitis. DSS, dextran sulfate sodium; DCs, dendritic cells; MLNs, mesenteric lymph nodes.

Accordingly, Pearson correlation analysis revealed a positive relationship between the neutrophil population in blood and the colonic bacterial load (**Figure 8A**, r = 0.631, p = 0.003), as well as between the percentage of HSP25 and HSP70 staining (**Figure 8B**, r = 0.443, p = 0.03). Furthermore, in the colon, the crypt HSP25 expression was negatively correlated with its bacterial load (**Figure 8C**, r = -0.511, p = 0.03) and the Ki67^+^ cell number (**Figure 8D**, r = -0.442, p = 0.03).

## Discussion

IBD is a recurrent GI disorder that affects a large and growing number of people in the world (1). Management of IBD has relied on anti-adhesion agents, anti-pro-inflammatory cytokine antibodies (e.g., anti-TNF antibody), and antibiotics. None of which could reverse the gut mucosa damages (2). Here, we first confirmed that DSS increased the expression of pro-inflammatory cytokines TNF-α (*Tnf*), *Il1β*, and *Ifng*, led to gut microbiota dysbiosis, and skewed immune responses in MLNs and the circulation, which in turn could increase intestinal permeability and bacterial translocation, consequently resulting in profound intestinal damage and colitis in young mice. More importantly, we demonstrate that peroral treatment with *L. reuteri* induces cytoprotective HSP70 and HSP25 expression in the colon of mice, ameliorates immunological disruptions, and modifies the DSS-shaped gut microbiome, thus strengthening the intestinal barrier function and protecting against colitis (**Figure 8E**). The strong association between epithelial HSP regulation and the concurrent probiotic effects against detrimental factors observed in this study provides novel insights into the natural defense mechanisms of gut epithelial cells and may open new avenues for IBD treatments.

A key determinant for intestinal homeostasis is maintaining its tolerance to the commensal microbiota while providing effective mechanisms to combat pathogens. In consistency with previous studies (5, 6, 24, 29), we found changes in the microbiota signature in the healthy intestine and colitis. DSS treatment recapitulates a loss of microbial α-diversity, which is often seen in IBD patients. It is an essential characteristic of a dysbiotic and less resistant gut microbiome, which may allow for pathogen invasion (30). In line with this, a significantly increased bacterial load in the colon of colitis mice was observed that was primarily driven by an overrepresentation of *Escherichia-Shigella*. Peroral treatment with *L. reuteri* successfully inhibited *Escherichia-Shigella* overgrowth and relieved the microbial load in the DSS-shaped microenvironment. In addition, DSS specifically increased the relative abundance of *Mucispirillum* in the colonic microbiota. As a mucus-foraging bacterium, it is likely that the abundance of *Mucispirillum* is negatively correlated with intestinal mucus integrity (31). In our previous study, *L. reuteri* treatment effectively ameliorates the DSS-resultant thinning effects on the colonic firmly adherent mucus layer in mice (4). In the current study, although *L. reuteri* treatment did improve barrier integrity and inflammatory responses, the colonic *Mucispirillum* in the *L. reuteri*-DSS group was not suppressed. Nevertheless, *L. reuteri* treatment restored the microbiota diversity and induced a unique bacterial profile during colitis, including a much higher level of *Alistipes* than that in the control and the DSS-treated mice. After a perturbation like DSS or antibiotics, gut microbiome may enter an alternative state, with various degrees of abilities to restore its equilibrium (30). We suggest that *L. reuteri* treatment not only offers colonization resistance against DSS-induced pathobiont enrichment but also favors symbiotic bacteria growth, therefore improving resilience of the gut microbial ecosystem. Finally, the microbiota dysbiosis may affect epithelial turnover (10). In the current study, we discovered a decrease in epithelium proliferation in the ileum, which may be attributed to the observed villi atrophy. In contrast, in the colon, cell proliferation was increased and was negatively linked with crypt HSP25 expression and further with the bacterial load. It could be argued that DSS-augmented bacterial pressure stimulated crypt hyperregeneration in the colon, which diluted HSP25 expression in the dividing crypt cells (32). Contrasting effects between ileum and colon were also observed in changes of gut microbiota in the current study, including the DSS-reduced ileal bacterial load. We speculate that it may be due to the much shorter transit time of the small intestine than that of the colon and based on the fact that the DSS model mainly manifests as colitis (33). This may also explain the less protective effects of peroral treatment with *L. reuteri* on the ileal bacterial community of DSS-treated mice.

The HSP functioning is tightly controlled in epithelial cells of the GI tract facing tremendous infectious and noninfectious threats (34). A commensal microbiota-driven intestinal HSP expression is suggested (20, 35), where an antibiotic treatment decreases mucosal HSP25 and HSP72 expression (20). In the current study, DSS-induced colitis significantly reduced the expression of HSP25 and HSP70 in the colon of young mice. Similarly, a defective induction of HSPs is seen in IBD patients (17). Peroral treatment with *L. reuteri* completely restored epithelial HSP expression in colitis mice, which subsequently coincided with an improvement in the epithelial barrier integrity visualized by ZO-1 and F-actin staining. Moreover, *Lactobacillus brevis* SBC8803 has been reported to confer health benefits in the intestine through HSP induction *in vitro* and *in vivo* (36). It seems that the balance of microbial community has a strong influence on HSP function, rather than the bacterial load itself. As highly conserved molecular chaperones, HSP25 and HSP70 work in concert and play multiple important roles in maintaining host homeostasis. They are both associated with the stabilization of the actin cytoskeleton of intestinal epithelium under stress conditions such as oxidative stress and inflammation (15). Furthermore, HSP25 and HSP70 have also been shown to regulate cell proliferation and apoptosis (37) by inhibiting actin polymorphism, preventing translocation of proapoptotic signals, or by inducing proper protein folding to promote cell survival (38). We dissected the HSP expression pattern in the current study and discovered that pretreatment with *L. reuteri* extends HSP70 and HSP25 along the villus/crypt axis in the colonic mucosa, with a stronger effect on HSP70. It suggests that under physiological conditions with an intact intestinal barrier, the signal of *L. reuteri* could reach the bottom of crypt cells to enhance HSP function. In contrast, DSS treatment with or without *L. reuteri* treatment aggregates the HSP defense at the mucosal surface, with a stronger effect on HSP25. It suggests that under inflammatory conditions, HSP25 may serve as the last resort against barrier disruption. In addition, we report that peroral treatment with *L. reuteri* reduces the bacterial load in MLNs and the expression of pro-inflammatory cytokines during colitis, in parallel with improved responses of conventional DCs and Foxp3^+^ T cells. This makes up one more event of epithelial HSP-mediated control of inflammatory signal cascades in colitis and with probiotic treatment. Interestingly, upregulation of HSPs in IECs is thought to directly inhibit increased levels of pro-inflammatory cytokines IL-6 and IL-8 in injured cells. While expression of HSP25/27 and HSP70 has been associated with enhanced anti-inflammatory cytokine IL-10 production in macrophages, DCs, and regulatory T cells, respectively (15, 39). It is also reported that overexpression of HSP27 gene facilitates the porcine epithelium to resist *Escherichia coli* infection *in vitro* (40).

Emerging evidence suggests that HSP70 and HSP25 exhibit various immunoregulatory features in gut homeostasis depending on the changes of the microenvironment (15). However, the exact modes of probiotic action in regulating epithelial HSPs in health and colitis were unclear. Here, we add to this by demonstrating that among the multiple mechanisms involved in the protection of *L. reuteri* treatment during experimental colitis, the inducible HSPs constitute an important defense system at the gut epithelial cells. DSS-induced gut microbiota dysbiosis may flare up skewed immune responses in MLNs and circulation. These, along with the increased expression of pro-inflammatory cytokines, and epithelial HSP reduction, indicate a loss of tolerance to perturbations, which in turn can promote intestinal permeability and bacterial translocation, leading to a detrimental positive feedback loop. The fact that peroral treatment with *L. reuteri* could manipulate interactions between gut microbiota, epithelial HSPs, and the immune system to restore the intestinal barrier function shows a tempting therapeutic potential for IBD treatment.

## Data Availability Statement

All data supporting the findings of this study are available within the article, in **Supplementary Information**, or from the corresponding author upon request. The bacterial 16S rRNA amplicon sequence data are available in European Nucleotide Archive database under accession number PRJEB12149 (ERP013591).

## Ethics Statement

The animal study was reviewed and approved by the Animal Care and Use Committee of the Yangzhou University (Permission No. YZUDWSY 2017-09-06).

## Author Contributions

H-YL, FG, and CuZ are the primary investigators in this study. H-YL, FG, CuZ, CZ, LY, MZ, and JY participated in the animal experiments. H-YL, PH, YZ, JD, and DC revised the article. WB and DC designed this study and wrote the article as corresponding authors. All authors contributed to the article and approved the submitted version.

## Funding

This work was supported by the National Natural Science Foundation of China (32002243), Natural Science Foundation of Jiangsu Province (BK20200932), Natural Science Foundation of the Higher Education Institutions of Jiangsu Province (20KJB230001), and the Priority Academic Program Development of Jiangsu Higher Education Institutions (PAPD).

## Conflict of Interest

The authors declare that the research was conducted in the absence of any commercial or financial relationships that could be construed as a potential conflict of interest.

## Publisher’s Note

All claims expressed in this article are solely those of the authors and do not necessarily represent those of their affiliated organizations, or those of the publisher, the editors and the reviewers. Any product that may be evaluated in this article, or claim that may be made by its manufacturer, is not guaranteed or endorsed by the publisher.
