# Supplementary material for: Epithelial Heat Shock Proteins Mediate the Protective Effects of Limosilactobacillus reuteri in Dextran Sulfate Sodium-Induced Colitis
Source: Front Immunol. 2022 Mar 7;13:865982. doi: 10.3389/fimmu.2022.865982 (PMC8934773; doi:10.3389/fimmu.2022.865982)
Supplement: Supplementary file 1 [file Table_1.docx]

**Supplementary information**

**Supplementary Table 1.**  Primers used for Real-Time qPCR.

| **Gene names and sequences** | |
| --- | --- |
| *Gapdh-F:* | *TGTGTCCGTCGTGGATCTGA* |
| *Gapdh-R:* | *CCTGCTTCACCACCTTCTTGAT* |
| *Il10-F:* | *TAAGGCTGGCCACACTTGAG* |
| *Il10-R:* | *GTTTTCAGGGATGAAGCGGC* |
| *Il1b-F:* | *GCAACTGTTCCTGAACTCAACT* |
| *Il1b-R:* | *ATCTTTTGGGGTCCGTCAACT* |
| *Il6-F:* | *TAGTCCTTCCTACCCCAATTTCC* |
| *Il6-R:* | *TTGGTCCTTAGCCACTCCTTC* |
| *Ifng-F:* | *TCAAGTGGCATAGATGTGGA* |
| *Ifng-R:* | *TCAAGTGGCATAGATGTGGA* |
| *Tnf-F:* | *CCCTCACACTCAGATCATCTTCT* |
| *Tnf-R:* | *GCTACGACGTGGGCTACAG* |
| *Tjp1-F:* | *AGGACACCAAAGCATGTGAG* |
| *Tjp1-R:* | *GGCATTCCTGCTGGTTACA* |
| *Ocln-F:* | *GCTGTGATGTGTGTGAGCTG* |
| *Ocln-R:* | *GACGGTCTACCTGGAGGAAC* |
| *HSPA1A-F:* | *ATGGACAAGGCGCAGATCC* |
| *HSPA1A-R:* | *CTCCGACTTGTCCCCCAT* |
| *HSPB1-F:* | *CGGAATTCATGGCCGAGCGCCGAGT* |
| *HSPB1-R:* | *CCGCTCGAGTTACTTGTTTTCCGGCTGTTCG* |
|  |  |
| *16S rRNA-F* | *GCAGGCCTAACACATGCAAGTC* |
| *16S rRNA-R* | *CTGCTGCCTCCCGTAGGAGT* |
| *Illumina adapter-N4-341-F* | *ACACTCTTTCCCTACACGACGCTCTTCCGATCTNNNNCCTACGGGNGGCWGCAG* |
| *Illumina adapter-805-R* | *AGACGTGTGCTCTTCCGATCTGACTACHVGGGTATCTAATCC* |

**The Western blot bands (related to Figure 4 E, F).**


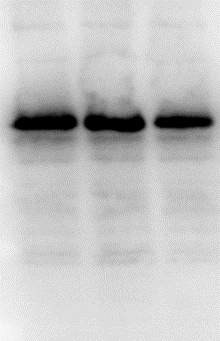


**100**

**70**

**35**

**25**

**HSP70**

**HSC70**


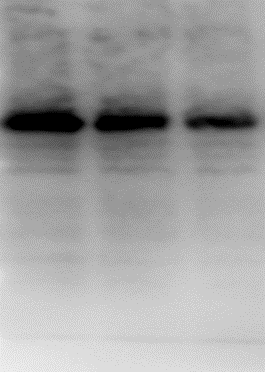

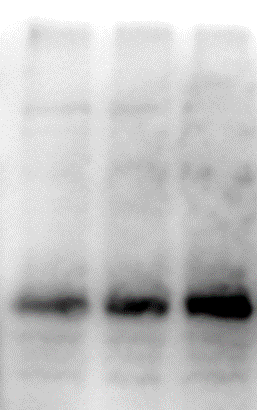


**100**

**70**

**35**

**25**

**HSP25**

**HSC70**


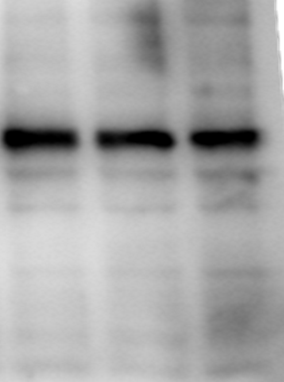

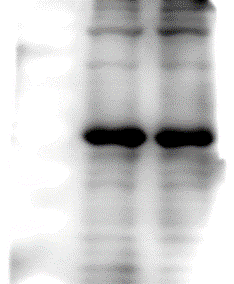

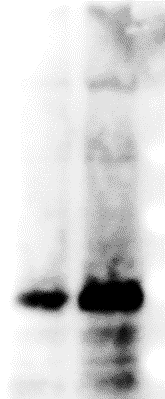


**100**

**70**

**35**

**25**

**HSP25**

**HSC70**

**HSP70**


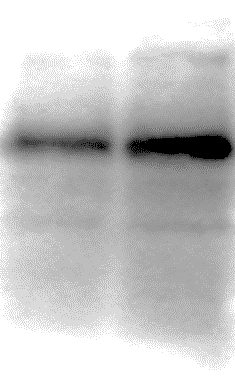

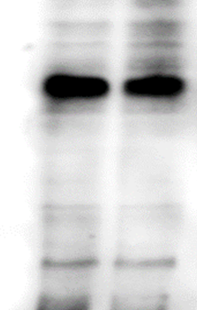


**HSC70**

**100**

**70**

**35**

**25**
